# The British Medical Association

**Published:** 1894-08-11

**Authors:** 


### The British Medical Association.

It was a happy thought which led the members of the British Medical Association to accept the invitation extended to them, and hold their sixty-second annual meeting at Bristol. Bristol is the metropolis of the West. It is noted for the hospitality of its inhabitants, and for the culture and standing of the members of the medical profession which minister to the needs of its population. Clifton Downs, the banks of the Avon, and the surrounding country, are delightful in scenery and aspect, and there are few places where the tired worker can more beneficially commence a holiday.

Dr. Long Fox is deservedly esteemed and beloved. He is the descendant of an old Bristol family which has sent many able men into the medical profession, and his genial presence and gentle bearing won for him golden opinions and unanimous acclamation. The professors of University College and the medical staff of the hospitals vied with each other to make the meeting produce a maximum of pleasure and satisfaction to the visitors both of high and low degree. Altogether it was rightly held that the Bristol meeting will be memorable in the annals of the Association, and that there has been nothing to compare with it since the meetings at Leeds and at Edinburgh. We have been requested, and very gladly take this opportunity, to tender to Dr. Long Fox, Dr. W. Johnstone Fyffe (treasurer), Dr. P. Watson Williams, the local editor of the *Daily Journal*, Dr. E. Markham Skerritt (the honorary local secretary), and to the chairmen, secretaries, and members of the local committees, individually and collectively, the grateful acknowledgments of the visitors, who felt personally indebted to one and all of them, and would gladly welcome an opportunity to give their feelings due expression. Dr. Skerritt must be especially congratulated, as he necessarily had the lion's share of the work, and his organising and administrative powers were placed beyond dispute by the excellence of the arrangements, which did not break down on any occasion or on any point, however small. The British Medical Association does much for the medical profession in many ways. We feel sure, however, that not the least useful of them all is the opportunity which its annual meetings afford to the members of a particular locality to develop faculties of administration which must prove highly beneficial to themselves and to the profession at large.

### The Business.

The annual general meeting affords members an opportunity of expressing their views as to the management of the Association on various points. The presentation of the annual report and the discussion which follows might be very well utilised for the purpose of directing attention to weak spots in the armour, and for pointing out directions in which the management might be strengthened or elaborated. The proceedings at the first general meeting on the Tuesday were scarcely worthy of the dignity of the greatest association known to the medical world. If we may judge those proceedings on their merits, we should incline to the opinion that the Council permits them to continue, year by year, because it holds, as we think wrongly, that they constitute a safety valve through which the discontented may discharge their venom and relieve their personal feelings. We are glad, however, to note as one result of the proceedings at Bristol, that the general body of members feel strongly that the time has arrived when due weight shall be attached to considerations which make for the dignity of the profession of medicine as represented by the discussions at its annual meetings. A great Association like this cannot dissociate itself from its members or the conduct of any portion of its work. The *British Medical Journal*, for example, is the very breath and life-blood of the Association, and, as we stated last week, it is an outrage to permit its editor to be baited by all and sundry, however unworthy may be the motives which lie at the bottom of the attack. The Association, of its own volition through its Council, deliberately underpays, and has deliberately underpaid its editor for very many years. Yet the sum expended upon literary contributions to its journal—some £6,000 per annum—places it within the power of the Council to give the editor £2,000 a year at least, and yet leave sufficient for casual contributors and others. If there is any just warrant for criticising the conduct of the journal, before the Council permit the loss of dignity suffered at Bristol to be repeated on a future occasion, it is their duty to look this question of the remuneratien of the editor in the face, and to settle it upon a basis which will warrant them in taking up a definite position in regard to the whole of the questions at issue. We hold that it is a function of the Congress to govern the Council of the Association, to be zealous for its honour, and to make such arrangements for the conduct of its business as shall protect both from discredit. Certain we are that unless something is done in the directions indicated, the loss of prestige which will result must be followed by a serious falling off in the number of members of the Association, as the dissatisfaction which so widely prevailed at Bristol is bound to find expression either by concerted action or by the retirement of individual members. The general business arrangements were excellent, and we congratulate Mr. Fowke, the general secretary, whose services, we are glad to note, are greatly appreciated by the members of Council. We spoke last week of Mr. E. Hart's eminent services as editor of the journal, and need not further emphasise them on the present occasion.

### Scientific Aspects of the Congress.

Dr. Osler, who is no mean authority, declared that the scientific aspects of the sixty-second annual meeting of the British Medical Association were more satisfactory and valuable than any of its predecessors. This satisfactory result is largely due to the zealous care and forethought of the President, Dr. Long Fox, who was deservedly popular. The discussions, which we hope to report in detail, were, on the whole, well sustained, the subjects were well chosen, and the attendance unusually good. The demonstrations given by Mr. Greig Smith and other members of the staff of the local hospitals, and in the Pathological Museum of University College, excited a great deal of interest, and helped to make the scientific aspects of the meeting very interesting and valuable.

The Museum.

A feature of these meetings which has grown imperceptibly to important dimensions in recent years is the annual museum. The catalogue of exhibits occupied nearly two hundred pages, and the exhibition itself contained much of surpassing interest and value, We purpose to give, in the course of the next few weeks, some account of certain special exhibits by Messrs. Allen and Hanbury, Down Brothers, Ferris and Co. (Bristol), Armour and Co., Alfred Carter, Corbyn, Stacey, and Co., Curry and Paxton, J. S. Fry and Sons, Jeyes' Sanitary Company, Lorimer and Co., Maltine Manufacturing Company (Limited), Mayer and Meltzer, Oppenheimer and Co., Park, Davies, and Co., Van Abbott and Sons, Vinolia, Wilson and Sons, Willows, Francis, and Butler, John Wyeth and Brother, A. and J. Warren, Christopher Thomas and Bros., Christy and Co., Giles, Schacht, and Co., Weiss and Son, Duncan, Flockhart, and Co., Evans, Lescher, and Webb, Godfrey and Cooke, Hertz and Collingwood, Horlick and Co., Krohne and Sesemann, Peat Industries Syndicate, Stephen Smith and Co., and some others. The exhibition was well organised, the exhibits had an exceptional interest, and visitors were more than usually well pleased and benefited by the time devoted to this branch of the Congress. The exhibition was divided into four sections—food and drugs, instruments, books, and sanitary and ambulance appliances. We have not space to speak of them in detail on the present occasion, but an early opportunity will be taken to call attention to those exhibits which are likely to prove of interest and value to readers of The Hospital.

Social Features of the Congress.

We have already alluded to the spirit of hospitality which so widely prevailed at Clifton. Dr. Long Fox gave a dinner party at the Queen's Hotel every evening throughout the session, and must have entertained quite 500 guests in this way alone. On Monday he entertained the members of Council and the distinguished guests who were present at the meeting. On Tuesday there were a great number of private dinners, followed by a conversazione at the Drill Hall, given by the exhibitors at the annual museum. On Wednesday the president, Dr. Long Fox gave a soirée at Clifton College, in connection with which the Zoological Gardens were illuminated, and an unusually beautiful display of fireworks took place. This entertainment alone must have cost Dr. Long Fox a very considerable sum. The Orpheus Union, of Bristol, sang some glees which attracted deserved applause and many hundred listeners. On Thursday the annual dinner took place in the large hall of the Victoria Rooms, and proved a marked success, the speeches being unusually good, Dr. Frederick T. Roberts, the cordiality of whose reception must have been very gratifying, being easily first. The arrangements for the dinner were so well carried out as to stamp Mr. J. Paul Bush as a caterer *par excellence*, and his ability in this direction has been already recognised by Bristol men. On Friday there was a Madrigal concert by the Bristol Society, followed by a ball at the Colston Hall. The guests were received by the Mayor and Mayoress, who laboured hard throughout the Congress to make the social arrangements successful; and, as Dr. Roberts put it, it was a sight for the gods to see the members of the British Medical Association dancing in hundreds. The Colston Hall was beautifully decorated, and had never looked so well in the memory of the oldest inhabitant. A delightful evening was spent by the visitors. In addition to these evening entertainments and receptions there were three garden parties, which were somewhat marred by the unfavourable weather; a luncheon by invitation of Mr. and Mrs. George Newnes, at the New Clifton Grand Pump Room, which Mr. Newnes has built; and a splendid luncheon at the Merchant Venturers' Hall, which everybody said was truly magnificent. The amount of private hospitality extended to members of the Congress was probably unequalled in its prodigality and kindliness. Not only were the visitors provided for by the local members of the profession, but the private residents vied with each other to promote the comfort and enjoyment of those members who were fortunate enough to be guests in private houses. The hotel accommodation, with the exception of the Clifton Downs and Royal Hotels, left much to be desired.

The Excursions.

On Friday a limited number of members were entertained at luncheon, at Bath, by the Mayor and Corporation, and in the afternoon a reception was given at the Pump Room, and the visitors were taken over the whole of the baths, including the Roman baths. Mr. H. W. Freeman, F.R.C.S.I., whose book on the Thermal baths of Bath has contributed much towards the popularity of these baths at the present time, was indefatigable, and his demonstation of the various appliances and of the treatment, left nothing to be desired. We believe that the completeness of these baths, the excellence of the arrangements, the results of the treatment, and the facilities afforded to invalids, surprised the majority of visitors at any rate. Everything possible has been done to restore the former popularity of these ancient bathing places, and it is matter for congratulation that English people are resorting there in increasing numbers every year. On the same day there were excursions to Clevedon and to the waterworks reservoirs, at Barrow Gurney, where Dr. D. S. Davies, the Medical Officer of Health, was indefatigable in his attention to the guests. On Saturday there were excursions to Berkeley Castle, by the kind invitation of Lord and Lady Fitzhardinge, and proved the most popular of them all; to Weston-super-Mare and Cheddar; to Wells and Glastonbury; to Lynmouth and Ilfracombe; and last, but by means least, to Chepstow Castle, Tintern Abbey, and Symond's Yat. All these excursions passed off without a hitch, everybody was more than satisfied, and they tended to cement the kindly feeling and happiness of the visitors to the British Medical Congress. One marked feature of these excursions was the cordiality of the local members of the British Medical Association, who gave up the day to enable them to act as guides to the various localities, and so to contribute greatly to the enjoyment and instruction of the visitors. Dr. Yeats gave a most interesting account of Chepstow Castle and Tintern Abbey, whilst Mr. Southall did the honours at Symond's Yat. We congratulate Dr. D. S. Davies (the chairman), Mr. J. Hancock Wathen (the secretary), and the members of the Excursion Sub-Committee, upon the complete success of all the arrangements; and everywhere there was abundant evidence to show how much brain power had been expended upon them, with the result that everything was well up to time, and excellently done besides. Dr. King's hospitality at Tintern Abbey was truly lavish, and his genial presence proved most grateful to the visitors. An interesting feature of the Tintern excursion was the reading of an unpublished letter of Thomas Carlyle's on a book entitled "The Healing Art, the Right Hand of the Church," by "Therapeutes," which we propose to deal with on another occasion. It is a somewhat remarkable coincidence that the subject matter of Thomas Carlyle's letter and of Canon Ainger's sermon in the Cathedral on Tuesday, the 31st ult., should have been identical, and some idea may be gained of the power and force of Canon Ainger's address from the leading article which appears in another column.

### ABSTRACTS AND NOTES OF ADDRESSES AND SECTIONS AT BRISTOL.

Our medical *confrères* at Bristol are to be congratulated on the scientific success of the meeting there last week, and we are confident that the sixty-second meeting of the Association will long be memorable for the advances in all branches of medicine and surgery resulting from the various discussions and contributions. The presidential addresses dealt generally with the advances made in the various departments of medicine and surgery in the past. We can only refer very briefly to a few points of special interest in one or two of them.

The address of the President, Dr. Edward Long Fox, is an excellent illustration of the value of "breadth of view in medicine," the theme of the opening remarks of the president of the medical section, Dr. F. T. Roberts. Dr. Fox referred to the difficulty that besets the giver of the presidential address, owing to the unwritten law of the Association which forbids any interference with subjects that might become matters of controversy in any of the sections, and which takes from the President the opportunity of devoting his remarks to a detailed consideration of any particular subject, but the Council has adopted a wise course in seeking from their president year by year an address which necessitates "breadth of view," and which can only be adequately undertaken by a master hand. We are, however, compelled to pass over the greater part of this valuable address as outside the scope of present article.

His remarks on the proper position of the medical profession in relation to the State were particularly happy. "What is the advantage of our numbers, what the use of our combinations, what the benefit of our knowledge of the wants of mankind, and of the modes of their relief, if we are not from time to time to assert our claims to be even more than we are at present—the advisers of the State? As a profession we are above party. Our highest aspirations tend to the formation of a pure commonwealth. The poor, the sick, and the criminal are our daily study, primarily for the relief of the individual, but with nobler and further-reaching aims, namely, that poverty may be diminished by the education of the nation in the wiser laws of health, by increased temperance, and by a knowledge from an early age of the common facts of physiology; and that the criminal class in the future may occupy narrower limits because no longer the victims of a debased heredity. . . . There are many here who can bear hearty witness to the value of that Local Government Board to the State. For the prevention of the spread of epidemic disease, for the cure of individual cases, for means of protection in town and country, for the use of all knowledge of microbal disease in the lower animals and in man." Our attention is directed to the recent developments in the care and treatment of feeble-minded children, and to the work of Dr. Güggenbuhl, who has led the way among the Swiss cretins as Pinet did among the idiots of France.

In his address on medicine Sir Thomas Grainger Stewart gave a very complete *résumé* of our knowledge of influenza, and this affection, which has caused more deaths and more dislocation of the social and commercial life of the nation than ever resulted from any cholera epidemic, is one of supreme importance. "We are now able positively to recognise its essential cause, by its presence or absence to test our diagnosis in cases otherwise doubtful, to judge better than formerly as to its mode of spreading, and with considerable confidence to explain many of its complications and sequelæ. For this discovery of the essential cause of the process we are indebted to Dr. R. Pfeiffer. Even to the naked eye influenza sputum is characteristic enough, of a yellowish greenish colour, viscid and tough, generally brought up in lumps or balls, and presenting a nummular appearance in the dish. The sputum varies in quantity from a very small amount up to several c.c. in 24 hours. In almost every case it is derived from the nose and pharynx, but also, in those of any severity, largely from the bronchial tubes, down to their utmost ramifications, and even from the air cells of the lungs. The special rodlets, the bacilli of influenza, which Pfeiffer separated from the numerous micro-organisms with which the mucous membrane of the healthy oral mucosa is infected, were found to be capable of cultivation only under special conditions, viz., when blood was present in the agar jelly of the culture medium Pfeiffer showed that in the blood it was the red corpuscles, not the leucocytes or the serum, that was the essential element for their growth, and that in the red corpuscles it was not the stroma but the hæmoglobin that was the necessary part; and that the value of the hæmoglobin did not depend upon the oxygen-absorbing power, but upon the iron it contained. Sir Thomas Stewart maintained that the bacillus produced the catarrhal symptoms directly, pointing out that the bodies of bacteria are capable of setting up inflammation and producing pus, as the experiments of Buchner have shown. We know that the bodies of the bacilli have the power of attracting white blood corpuscles towards them by positive chemotaxis, as was shown in the experiments of Gabribchewsky. This is typically seen in gonorrhœa. But when we come to the consideration, not of the local but the general constitutional effects — the degrees of fever, the aching pains, the general debility, which are ordinary symptoms of the malady—these could not be ascribed to the direct action of the poison, but rather to that of their products.

What measures should be adopted to prevent the spread of influenza? Outside the body of the healthy person the bacillus might be prevented from producing the effects if we could effectively isolate those who have become affected with the disease, but isolation in a general way is impossible, and that because many cases of true influenza are so mild as to escape detection, and because even where the case is severe infection is often spread before diagnosis is possible. But now that the general public more properly appreciate the dangers of influenza, precautions are more often taken, and thus people suffering from heart or any disease have been kept scrupulously away from the infected, and have thereby escaped what would have been to them a terrible danger. But even where isolation is impossible much good may be done by destruction of the sputum and of the nasal secretion. So precautions as to the discharge if once efficiently taken would tell materially upon the epidemics, and so sensitive are the bacilli that any antiseptic ordinarily in use will probably suffice. Inhalation of such remedies as eucalyptus and menthol have been widely employed, but the lecturer himself suggested that much good might be expected from intratracheal injections, such as 10 per cent. solution of menthol in olive oil, with or without 2 per cent. of guaiacol superadded."

Dr. A. T. Harrison, in the section of dermatology, drew attention to the therapeutic value of rest in skin affections. Many skin affections keep the patient confined to bed for a longer or shorter time as a matter of course, but there are many cases of severe eczema and extensive psoriasis which permit patients to be up and about, either because they cannot afford the time to rest or have not the inclination for it; and yet if we confine these patients in bed for awhile, and the same treatment, so far as applications and drugs are concerned—which produced only limited or slow effects—will now act thoroughly and rapidly, and the results are altogether very surprising and satisfactory. The effect of rest in treatment is especially evident when the nervous system begins to flag, and sleep is broken and the appetite and digestion impaired.

Mr. Greig Smith, in his address on surgery, dwelt on the art of surgery and how we train men to practice it, and the views he expressed are worthy of a great surgeon ever seeking to raise surgery, and our conception of what should constitute the type of a surgeon, to a higher platform.

Dr. Frederick T. Roberts, in speaking on breadth of view in medicine, reminded us of the tendencies which prevail amongst the laity of the present day, and which, he believed, are even now doing much harm, and likely to lead to much more serious mischief in the future if not checked, viz., the tendency to regard each system and organ in the body as a distinct entity, working independently of all the rest, while their relation to general conditions of the body are often ignored; and to attribute every disorder or symptom, whatever may be its nature, invariably to one particular organ, which differs according to the peculiar fad of the individual. He was not at all sure that the profession is altogether free from these tendencies, or, at any rate, that we realise to their full extent the pathological relations of organs and systems to each other, or adequately recognise their connections with various general morbid conditions.

Dr. G. F. Blandford, in his address on psychology, urged the importance of practical measures for the prevention of insanity, and this chiefly by limiting the propagation of that most fearful disease through the union of affected persons. Although we cannot forbid all children of an insane stock to marry, it is most important that they should marry only those who are themselves free from all nervous disorders, and whose inheritance is untainted by neuroses. He looked to a higher sense of responsibility on these points on the part of the public as the only means by which these desirable ends could be attained, and the education of public opinion, he reminded us, rests mainly with medical profession.

Dr. Dickinson addressed the section of diseases of children on "Some of the characters of diseases of childhood." He emphasised the necessity of plenty of food and the special need of warmth in young children. He had often thought that schoolboys were not fed well enough. As regards alcoholic drinks, the younger the child the more harm they did. Respiratory affections accounted for a large percentage of the deaths in childhood, and children are more immediately affected by aerial influences, whether good or bad, than are grown people. Thus pure air, especially sea air, is our greatest remedy in the chronic ailments of childhood.

In introducing the discussion on *Functional Diseases of the Heart*, Dr. Douglas Powell defined functional disease of the heart as "a perverted action and sensibility of the heart, not dependent on structural changes." Under cardio-vascular hyperæsthesia three varieties may be distinguished, (*a*) those in which there is simply undue consciousness of the heart's action; (*b*) conditions in which this consciousness is associated, sometimes with high blood pressure, with more or less violent attacks of cardiac disorder, headache, a sense of impending disaster; (*c*) the more serious group of cases in sensitive neurotic persons, in whom very alarming symptoms may be precipitated by emotion, visceral irritation, or surface chill. While clinically these patients have usually a high tension pulse during the attacks, inconstancy of tension is the rule in these cases in the intervals between the attacks, with mental alarm and general restlessness. The treatment he advocated for these classes of cases consisted in (1) the administration of cardio-vascular sedatives; (2) the removal of high tension; and (3) the use of digitalis and drugs of that class.

In regard to tachycardia, he wished carefully to exclude all cases in which rapid heart action was associated with symptoms of graver disease. The main features in tachycardia are (*a*) persistent hurry of the pulse, lasting for periods varying from hours to many weeks, during which the pulse is frequent, regular, small, with sustained rather than high tension; (*b*) intervals of severe palpitation, attended with precordial pain, notable distress, and a tumultuous action of the heart; (*c*) a tendency to abrupt onset and equally sudden decline in the attack; (*d*) the exciting causes are mental shock and mental or physical prostration. Dr. Powell drew attention to the difficulty in distinguishing between the cases of graver diseases in which the heart disturbance is only one symptom, and those in which it is the manifestation.

In bradycardia, on the other hand, the pulse is slow and there may be intermediate heart beats, and this pulse he had noticed after diphtheria, the acute fevers, and in association with excess of urea in the urine. The two sources of this affection are (*a*) neuritis of the cardiac nerves; (*b*) a partial inhibition of the cardiac muscle by a morbid condition, *e.g.*, undue acidity of the blood.

